# Cue avoidance training and inhibitory control training for the reduction of alcohol consumption: a comparison of effectiveness and investigation of their mechanisms of action

**DOI:** 10.1007/s00213-017-4639-0

**Published:** 2017-05-27

**Authors:** Lisa C. G. Di Lemma, Matt Field

**Affiliations:** 1 Department of Psychological Sciences, University of Liverpool, L69 7ZA, Liverpool, UK; 2 The UK Centre for Tobacco and Alcohol Studies, Liverpool, UK

**Keywords:** Alcohol, Cognitive bias modification, Devaluation, Inhibitory control

## Abstract

**Rationale:**

Both cue avoidance training (CAT) and inhibitory control training (ICT) reduce alcohol consumption in the laboratory. However, these interventions have never been directly compared and their mechanisms of action are poorly understood.

**Objectives:**

We compared the effects of both types of training on alcohol consumption and investigated if they led to theoretically predicted changes in alcohol avoidance (CAT) or alcohol inhibition (ICT) associations and changes in evaluation of alcohol cues.

**Methods:**

Heavy drinking young adults (*N* = 120) were randomly assigned to one of four groups: (1) CAT (repeatedly pushing alcohol cues away with a joystick), (2) sham (control) CAT; (3) ICT (repeatedly inhibiting behaviour in response to alcohol cues); or (4) sham (control) ICT. Changes in reaction times and automatic evaluations of alcohol cues were assessed before and after training using assessment versions of tasks used in training and the implicit association test (IAT), respectively. Finally, participants completed a bogus taste test as a measure of ad libitum alcohol consumption.

**Results:**

Compared to sham conditions, CAT and ICT both led to reduced alcohol consumption although there was no difference between the two. Neither intervention affected performance on the IAT, and changes in reaction time did not suggest the formation of robust alcohol avoidance (CAT) or alcohol inhibition (ICT) associations after training.

**Conclusions:**

CAT and ICT yielded equivalent reductions in alcohol consumption in the laboratory. However, these behavioural effects were not accompanied by devaluation of stimuli or the formation of alcohol avoidance or alcohol inhibition associations.

**Electronic supplementary material:**

The online version of this article (doi:10.1007/s00213-017-4639-0) contains supplementary material, which is available to authorized users.

## Introduction

According to dual-process models of addiction, loss of control over substance use arises from conflict between two partially independent systems: a fast ‘impulsive’ system that is triggered by automatic appetitive responses to substance-related cues and a slower ‘reflective’ system that is dependent on the integrity of executive functions which are weakened by chronic substance use and exposure to substance-related cues (Wiers et al. 2007; Hofmann et al. 2009; Gladwin and Figner 2014; McClure and Bickel 2014).

Regarding automatic processes, there is compelling evidence that alcohol-related cues evoke automatic approach tendencies. The strength of these tendencies can be assessed with the approach avoidance task (AAT; Wiers et al. 2009) or related tasks (Field et al. 2008). For example, during the AAT, participants are instructed to ‘approach’ or ‘avoid’ alcohol or control pictures by moving a joystick towards or away from them. A number of studies with non-dependent drinkers have confirmed that, compared to light drinkers, heavy drinkers are faster when required to approach rather than avoid alcohol-related pictures (see Kersbergen et al. 2015; Watson et al. 2012).

Regarding reflective processes, heavy drinkers have impaired executive functions, including the ability to inhibit behaviour (Smith et al. 2014). Furthermore, alcohol-related cues may exacerbate these deficits (Jones and Field 2015; Petit et al. 2012). Inhibitory control is typically assessed with computerized tasks such as the Go/No-Go and Stop-Signal tasks, both of which require participants to respond rapidly but inhibit responding when infrequent ‘stop’ or ‘no-go’ signals are presented (Verbruggen et al. 2014). A recent meta-analysis demonstrated that heavy drinkers perform poorly on these tasks, and this effect is robust across studies (Smith et al. 2014). Other studies have demonstrated that the presence of alcohol-related cues impairs inhibitory control among alcohol consumers (Petit et al. 2012; Jones and Field 2015).

Dual-process models have implications for the prevention and treatment of addiction. Specifically, the aim of ‘cognitive bias modification’ (CBM) is to extinguish or reverse the aforementioned cognitive biases in order to reduce drinking behavior (Wiers et al. 2013; Gladwin et al. 2016). For example, in cue avoidance training (CAT; Wiers et al. 2011), participants practice making avoidance movements in response to alcohol-related cues, whereas in inhibitory control training (ICT; Houben et al. 2011) participants practice inhibiting their behaviour in response to alcohol cues. The aim of both types of CBM is to alter participants’ alcohol-related automatic associations so that alcohol cues will evoke more adaptive responses when they are encountered after CBM. Development and initial evaluation of CBM interventions typically begins with laboratory studies which investigate the effects of a brief ‘dose’ of CBM on a behavioural measure of the motivation to drink (such as a bogus ‘taste test’; see Jones et al. 2016), in comparison to a matched control intervention. If these laboratory studies suggest that CBM can reduce the motivation to drink, this provides strong justification for evaluating the effectiveness of multiple sessions of CBM in clinical populations, ideally using randomized controlled trials (RCTs) (Allom, Mullan and Hagger 2015; Gladwin, Wiers and Wiers 2016; Jones et al. 2016; Kakoschke et al. 2017a).

Laboratory studies of CAT (see Kakoschke et al. 2017a) have demonstrated that a single session of this intervention strengthens alcohol-avoidance associations and reduces alcohol consumption, among non-dependent heavy drinkers (Wiers et al. 2010; Sharbanee et al. 2014). Subsequent trials of CAT with alcohol-dependent patients demonstrated a reduced likelihood of relapse after CAT (compared to a control intervention; Wiers et al. 2011; Eberl et al. 2013; Gladwin et al. 2015; Manning et al. 2016). These effects of CAT on drinking outcomes were mediated by changes in alcohol-avoidance associations in some of these clinical studies (Wiers et al. 2011; Eberl et al. 2013; Gladwin et al. 2015), although this was not observed in a more recent study (Manning et al. 2016). Similarly, several studies have demonstrated that a single session of ICT leads to reduced alcohol (or food) consumption in the laboratory (relative to a control intervention), and two recent meta-analyses of these findings have confirmed that this effect is small but robust across studies (standardized mean difference (SMD) = 0.43 in Jones et al. (2016); and SMD = 0.38 in Allom, Mullan and Hagger (2015)). There is also some evidence that these effects may persist to influence drinking outside of the laboratory (see Allom, Mullan and Hagger, 2015), although to date there are no published trials that investigated the effectiveness of multiple sessions of ICT for alcohol-dependent patients.

Despite these promising effects on drinking behavior in the laboratory and outcomes after treatment, more research is needed to clarify the mechanisms of action of CBM. The most parsimonious explanation is that avoidance (and inhibition) can be associatively mediated, such that repeatedly avoiding motivationally salient cues, or refraining from responding when exposed to those cues, leads to the formation of stimulus-avoidance (CAT) or stimulus-stop associations (ICT), respectively. Subsequently, these learned associations should manifest as automatic avoidance or inhibition when those cues are next encountered (Verbruggen, McLaren and Chambers 2014). The findings discussed above regarding the formation of alcohol-avoidance associations after CAT, and their importance as mediators of effects of CAT on drinking behavior, are consistent with this view (see Gladwin, Wiers and Wiers 2016; Kakoschke et al. 2017a). Regarding ICT, numerous studies have demonstrated the formation of ‘stopping’ associations (inferred from slowing of reaction times) when arbitrary cues are paired with inhibition of behavior (Verbruggen and Logan 2008a, 2009; Chiu and Aron 2014; Best et al. 2015; Bowditch, Verbruggen and Mclaren 2016; Houben and Jansen 2015). In our recent meta-analysis of applied studies, we demonstrated that failures to inhibit during ICT diminished the effect of ICT on eating and drinking behaviour in the laboratory, presumably because each inhibition failure weakens the association between target cues and successful inhibition (Jones et al. 2016).

Formation of automatic alcohol-avoidance or alcohol-inhibition associations may ultimately lead to changes in drinking behavior through a shared mechanism, namely both types of associations may lead to devaluation of alcohol-related cues, which in turn may blunt the ability of those cues to influence behaviour (see Guitart-Masip et al. 2014; Veling, Holland, and van Knippenberg 2008). A number of studies have demonstrated that stimuli paired with inhibition of behaviour (Veling, Aarts and Papies 2011; Ferrey, Frischen and Fenske 2012; Veling, Aarts and Stroebe 2013; Wessel et al. 2014) or overt avoidance responses (Kemps et al. 2013; Schonberg et al. 2014; Woud et al. 2013b) are evaluated more negatively than stimuli paired with behavioural responding or overt approach, respectively. Particularly relevant here are findings from two studies which demonstrated that a reduction in alcohol consumption after a single session of ICT was accompanied by changes in automatic evaluations of alcohol pictures, which became more negative after ICT (Houben et al. 2011, 2012).

To our knowledge, no previous study has contrasted the effects of CAT and ICT on alcohol consumption in the laboratory, or investigated if both interventions yield equivalent changes in devaluation of alcohol-related cues. The primary aim of the present study was to investigate if both CAT and ICT would be equally effective at reducing alcohol consumption, relative to appropriate control groups (‘sham’ training conditions which apply a 50% contingency; Kakoschke et al. 2017a). Our secondary aim was to investigate if these interventions would lead to the development of alcohol-avoidance (CAT) or alcohol-inhibition (ICT) associations, and if changes in these associations would be accompanied by equivalent changes in automatic evaluations of alcohol-related cues.

## Method

### Participants

One hundred and twenty (86 females, 34 males) heavy drinkers were recruited from staff and students at the University of Liverpool via online and poster advertising. Inclusion criteria included average weekly alcohol consumption in excess of the United Kingdom Department of Health guidelines (at the time, these were 14 and 21 units per week for females and males, respectively; note that these guidelines were revised in January 2016, after completion of this study). Participants were also required to be aged between 18 and 25, fluent in English, have normal or corrected to normal vision and no history of alcohol use disorders. The study was approved by the University of Liverpool Research Ethics Committee.

### Design

A mixed design was employed. Participants were randomly assigned to one of four groups (using an online random number generator) that reflected the between-subject factors of training type (CAT or ICT) and condition (active training or sham training). The within-subject factor was time because assessment tasks (IAT, AAT and GNGT) were administered before the training (pre-test) and afterwards (post-test).

### Materials

Computer tasks were presented on a Dell desktop computer with a 15″ monitor. Participants responded using a standard keyboard and a joystick. Tasks were programmed and administered in Inquisit version 3.0 [Millisecond Computer software] (2009).

Twenty pairs of alcohol-related and matched neutral (control) pictures were used in the computer tasks (Field et al. 2004; Barkby et al. 2012). Alcohol pictures depicted alcoholic drinks (e.g. bottles or glasses) and drinking scenes (e.g. models holding a beverage or drinking it) and each was matched to a neutral picture that depicted stationery (e.g. pens, staplers) and models using those items (e.g. holding pens or stapling paper).

### Approach avoidance task and cue avoidance training (based on Wiers et al. 2010).

During each trial, an alcohol-related or control picture was presented in the centre of the screen, and participants were required to rapidly categorize pictures according to their spatial orientation (landscape or portrait), but to ignore the content of the pictures. Participants were instructed to ‘approach’ pictures presented in one format (e.g. portrait orientation) by pulling the joystick towards them and ‘avoid’ pictures presented in the other format (e.g. landscape orientation) by pushing the joystick away. During each trial, the picture remained on screen until the participant responded or until a 1000 ms timeout had elapsed. Correct approach responses caused a zooming effect (the picture became larger), and correct avoidance responses caused a shrinking effect (the picture became smaller). Incorrect responses or failure to respond in time led to error feedback in the form of a red cross displayed in the centre of the screen for 500 ms.

The task comprised four blocks: a brief practice block (10 trials), a pre-test assessment block (80 trials), the cue avoidance or sham training block (480 trials, with a short break half-way through) and a post-test assessment block (80 trials). Participants were not informed when the task switched between assessment and training blocks. Picture format was counterbalanced, with half of participants instructed to pull landscape and avoid portrait format pictures, and reversed instructions for the remaining participants. Participants were required to make an equal number of push and pull responses in all blocks. Trial order within each block was randomized.

The pre-test and post-test assessment blocks were identical, and each contained 50% alcohol and 50% control pictures, half of each in portrait format and half in landscape format. In these blocks, participants had to approach and avoid alcohol and control pictures with equal frequency. In the training block (in which only a subset of 10 of the alcohol-related and 10 matched control pictures were used; see supplementary materials), for participants in the active training group 90% of alcohol pictures were presented in the format requiring an avoidance movement, whereas 90% of control pictures were presented in the format requiring an approach movement. For participants in the sham training group, 50% of both alcohol and control pictures were presented in the format requiring an avoidance movement with the remainder requiring an approach movement.

### Go/no-go task and inhibitory control training (based on Houben et al. 2012).

During each trial, an alcohol-related or control picture was presented in the centre of the screen with one of two letters (‘p’ or ‘f’) superimposed on one of the four corners of the picture. Participants were instructed to press the space bar if the go cue (‘p’) was present, but to withhold their response if the no-go cue (‘f’) was present. During each trial, the picture and letter remained on screen until the participant responded or until a 1500 ms timeout had elapsed. Feedback was presented on each trial: a centrally presented green circle (500 ms) for correct responding (pressing the spacebar before the 1500 ms timeout on Go trials, and successfully withheld responses on no-go trials), and a red cross (500 ms) for incorrect responding (omission errors on go trials and commission errors on no-go trials).

The task comprised four blocks: a brief practice block (10 trials), a pre-test assessment block (80 trials), the inhibitory control or sham training block (480 trials, with a short break half way through) and a post-test assessment (80 trials). Participants were not informed when the task switched between assessment and training blocks. Trial order within each block was randomized.

The pre-test and post-test assessment blocks were identical. Each contained 50% alcohol and 50% control pictures, half of each accompanied by go and no-go cues; therefore, participants had to respond and inhibit to alcohol and control pictures with equal frequency. In the training block (in which only a subset of 10 of the alcohol-related and 10 matched control pictures were used; see supplementary materials), for participants in the active training group, 90% of alcohol pictures were accompanied by no-go cues, whereas 90% of control pictures were accompanied by go cues. For participants in the sham training group, 50% of both alcohol and control pictures were accompanied by no-go cues, and the remainder was accompanied by go cues.

### Pictorial implicit association task

We adapted a bipolar alcohol valence IAT (described in Houben et al. 2012), which is a categorization task that assesses the strength of associations between alcohol pictures and valenced words. Participants were instructed to rapidly categorize stimuli into two target categories (alcohol or stationery) and two attribute categories (positive or negative valence), by responding with one of two different response keys. The rationale is that participants should be faster to categorize targets and attributes that are strongly associated (e.g., alcohol pictures and positively valenced words) during blocks of the task in which the target and attribute share a response key. A complete description of the task is provided in supplementary materials.

### Procedure

**Fig. 1:**
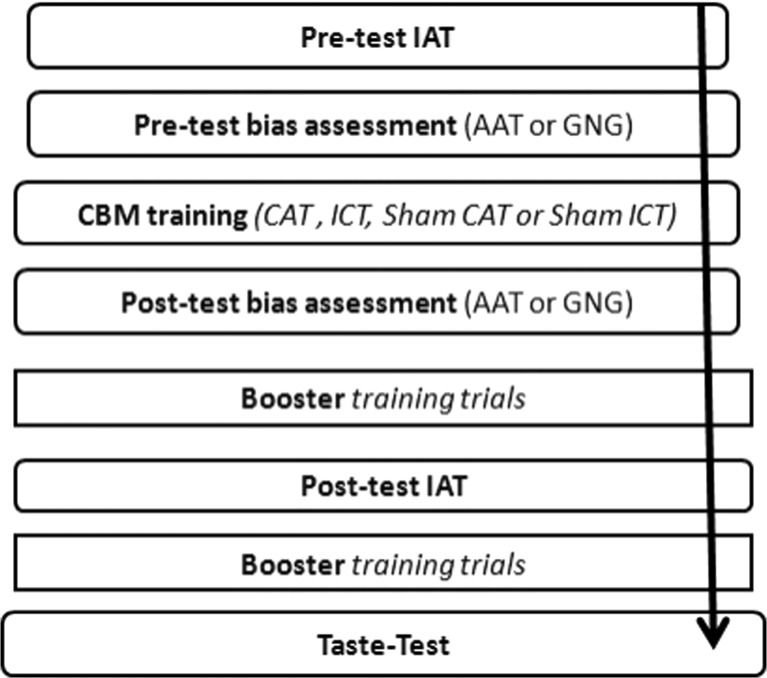
Schematic overview of the experimental procedure. See “Method” section for details

Participants were advised that the aim of the study was to investigate relationships between cognitive performance and individual differences in drinking habits (Fig. 1). Testing sessions took place between 12:00 and 19:00 in a quiet laboratory. Participants provided informed consent and a breathalyzer reading (all participants had a breath alcohol content of zero), before being seated at a desk approximately 1 m away from the computer monitor. Participants completed the pre-test IAT followed by the pre-test AAT or GNG task (depending on group allocation). They then completed the training block of the CAT or ICT before immediately completing the post-test assessment (AAT or GNG task). Participants then completed an additional 80 ‘booster’ CAT or ICT training trials (with the same contingencies that were applied during the training block) before completing the post-test IAT. They then completed a further 80 ‘booster’ trials before completing the alcohol taste-test: four chilled drinks (200 ml each) were presented simultaneously: beer (Fosters, 4% alcohol by volume (ABV)), cider (Magners original, 4.5% ABV) and two soft drinks (Coca Cola and Fanta Orange). Participants were instructed to rate and rank each drink on 10 different characteristics (e.g. fruitiest, sweetest and fizziest; see Jones et al. 2011), and they were informed that they could “*drink as much or as little as they liked in order to give a valid answer to the questions*”. After 10 min had elapsed, the drinks were removed and the volume of each drink consumed was recorded, out of sight of the participant.

Participants then provided general demographic information and completed the following battery of questionnaires: a 2-week timeline follow-back retrospective alcohol diary (TLFB; Sobell and Sobell 1992), the Alcohol Use Disorders Identification Test (AUDIT; Saunders et al. 1993), the Temptation and Restraint Inventory (TRI; Collins and Lapp 1992), the Contemplation Ladder (CL; Biener and Abrams 1991; Hogue, Dauber, and Morgenstern 2010) and the Readiness to Change Questionnaire (RTCQ; Rollnick, Heather, Gold, and Hall 1992). Participants’ awareness of the experimental hypotheses was assessed using a funneled debriefing self-report measure adapted from previous studies (Jones and Field 2013). We assessed participants’ beliefs about the general aims of the experiment, and their awareness of the purpose of the training and the taste-test; the wording of questions and participants’ responses is reported in supplementary materials. Half of the participants in each group completed the awareness check before the questionnaire battery. At the end of the experiment, participants were debriefed, breathalyzed and compensated either with course credits or shopping vouchers (£15 Sterling).

### Data processing prior to analysis

For the IAT*,* we computed the *d-measure* (Greenwald, Nosek and Banaji 2003), which indicates the strength of associations between alcohol and positive versus negative words. See supplementary materials for details. In order to investigate changes in cue-approach and cue-inhibition associations in the AAT and GNG tasks after CAT and ICT, respectively, we first excluded trials with errors and those with outlying reaction times (faster than 200 ms or slower than 2000 ms, then those that were more than 3 SDs above the mean) before comparing reaction times on each trial type at pre-test and post-test assessments.

## Results

### Group characteristics

All variables in Table 1 (with the exception of gender ratio) were analyzed using univariate ANOVAs with a between-subject factor of group (4: active CAT, sham CAT, active ICT, sham ICT). After Bonferroni correction to account for multiple contrasts, there were no significant group differences on any of these variables (Fs < 3.03, ps > .03). There were more female than male participants in all groups, and this gender imbalance was particularly pronounced in the sham CAT and active ICT groups (*Χ* ^2^(3) = 8.37, *p* = .04).

**Table 1:** Group characteristics

	CAT	Sham CAT	ICT	Sham ICT
Age (years)	20.37 (2.14)	20.40 (2.09)	20.07 (1.95)	20.43 (1.87)
Gender ratio (M/F)	11:19	5:25	5:25	13:17
Weekly alcohol consumption	24.14 (10.63)	24.72 (9.98)	24.43 (13.78)	26.70 (11.00)
AUDIT	14.60 (6.21)	13.23 (3.99)	13.40 (5.84)	14.47 (5.65)
Contemplation ladder	3.33 (2.50)	2.37 (2.50)	3.03 (2.40)	3.77 (2.92)
TRI concern	7.10 (4.75)	5.37 (2.82)	6.33 (3.04)	7.27 (4.40)
TRI restrict	9.97 (5.40)	7.53 (4.18)	8.33 (4.06)	10.80 (4.98)
TRI govern	10.17 (6.63)	7.10 (4.50)	8.30 (4.73)	10.50 (4.89)
TRI emotion	10.30 (5.47)	8.70 (5.49)	9.20 (4.10)	11.27 (6.03)
TRI cognitive preoccupation	5.73 (3.09)	5.33 (3.25)	5.03 (2.57)	6.63 (3.32)
TRI concern about drinking	7.10 (4.75)	5.37 (2.82)	6.33 (3.04)	7.27 (4.40)
RTCQ pre-contemplation	0.00 (3.41)	0.67 (3.05)	0.37 (3.45)	−1.30 (3.63)
RTCQ contemplation	−0.40 (4.55)	−1.90 (3.12)	−0.93 (3.08)	0.33 (3.99)
RTCQ action	−3.70 (3.27)	−4.23 (4.14)	−3.67 (3.22)	−3.00 (4.22)

Values are mean ± SD

*Weekly alcohol consumption* = self-reported typical weekly alcohol intake, in UK units. *AUDIT* = Alcohol Use Disorders Identification Test, values range from 0 to 40. *TRI* = Temptation and Restraint Inventory subscales range from 3 to 27; *RTCQ* = Readiness to Change Questionnaire subscales range from −8 to +8. Contemplation Ladder is a 10-point Likert scale (0 = no willingness to change; 10 = taking action to change)

### Effects of training on alcohol consumption

**Fig. 2:**
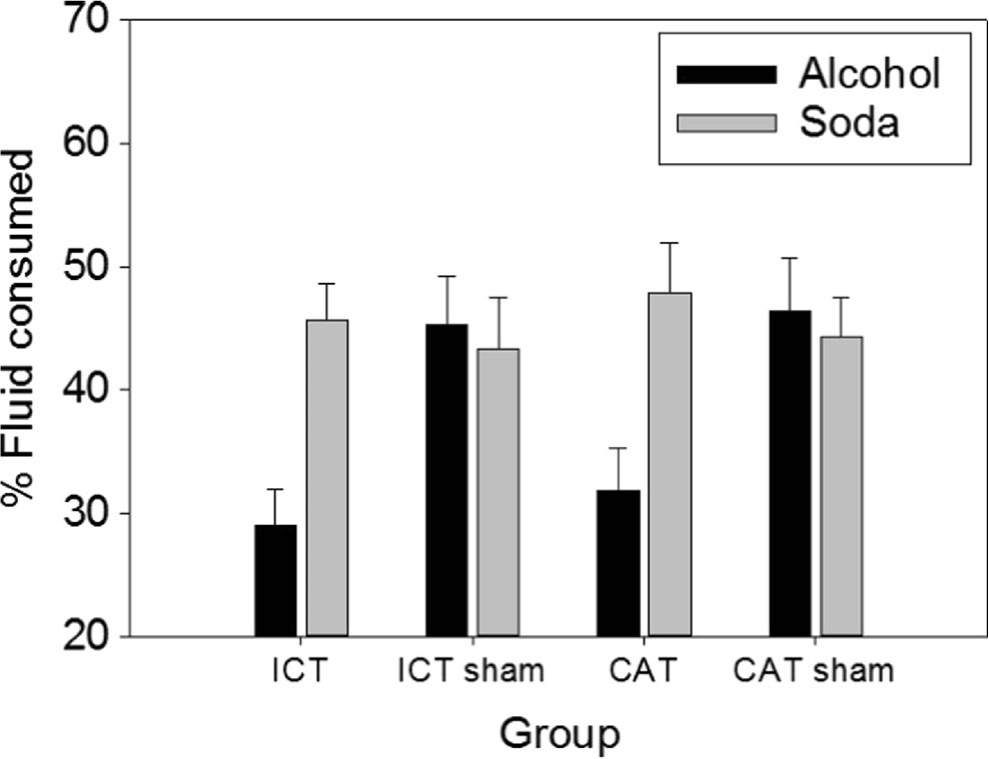
Alcohol and soda consumption during the taste test, calculated as a percentage of the total volume of each type of fluid available, separated by training groups. Values are means (*error bars* indicate SEM)

Alcohol and soft drink consumption was calculated as a percentage of the total volume of each type of fluid available (Fig. 2). Group differences in alcohol and soft drink consumption were analyzed using a 2 × 2 × 2 mixed design ANOVA, with a within-subject factor of drink type (2: alcohol, soft drink) and between-subject factors of training type (2: CAT or ICT) and condition (2: active training, sham training). Results revealed a statistically significant main effect of drink type (*F*(1116) = 15.75 *p* < .01) that was subsumed under a significant drink type × condition interaction (*F*(1116) = 26.08, *p* < .01).

Participants in the active training conditions consumed less alcohol (*M* = 30.39%, *SD* = 17.67), than participants in the sham training conditions (*M* = 45.86%, *SD* = 22.06). This difference was significant, *t*(118) = 4.24, *p* < .01; representing a medium to large effect size (Cohen’s *d* = .78). However, there were no significant differences in soda consumption between the active training conditions (*M* = 46.76%, *SD* = 19.16) and the sham training conditions (*M* = 43.80%, *SD* = 20.04; *t*(118) = .83, *p* = .41; *d* = .15). Importantly, the three-way interaction between drink type, training type and condition was not significant (*F*(1116) = .01, *p* = .94). Therefore, both types of training (CAT and ICT) were equally effective at reducing alcohol consumption.

### Reaction times before and after cue avoidance training

Approach and avoidance RTs were subjected to a 2 × 2 × 2 × 2 mixed design ANOVA, with within-subject factors of time (2: pre-test, post-test), picture type (2: alcohol, control), movement (2: approach, avoidance) and a between-subject factor of condition (2: active training, sham training) (Table 2). The main effect of movement was statistically significant (*F*(1,58) = 19.31 *p* < .01), reflecting faster RTs to initiate approach rather than avoidance movements. The hypothesized four-way interaction time × picture type × movement × condition approached significance (*F*(1,58) = 3.47 *p* = .07), and there were no other significant main effects or interactions (Fs < 2.53, ps > .12).

**Table 2:** Reaction times (milliseconds) to approach and avoid alcohol and control pictures during the approach-avoidance task (AAT)

	Active training	Sham control
*Pre-test*		
*Approach alcohol*	*758.07 (147.68)*	*743.76 (128.53)*
*Avoid alcohol*	*799.10 (162.06)*	*763.97 (117.97)*
*Approach control*	*772.22 (169.59)*	*769.85 (143.04)*
*Avoid control*	*789.35 (166.34)*	*771.38 (133.15)*
*Post-test*		
*Approach alcohol*	*754.96 (135.66)*	*748.45 (148.04)*
*Avoid alcohol*	*767.10 (134.64)*	*797.80 (185.41)*
*Approach control*	*749.20 (133.61)*	*764.13 (177.99)*
*Avoid control*	*782.91 (150.91)*	*781.74 (178.17)*

Values are shown separately for active training and sham control training groups, and at pre-test (before cue avoidance training) and post-test (after cue avoidance training). Values are mean ± SD

We explored this interaction by running three-way ANOVAs separately on the data at pre-test and post-test. At pre-test, the picture type × movement × condition interaction was not significant (*F*(1, 58) = .05, *p* = .82). However, there were main effects of picture type (*F*(1, 58) = 4.17, *p* = .05) and movement (*F*(1, 58) = 6.63, *p* = .01), which were subsumed under a picture type × movement interaction, which approached significance (*F*(1, 58) = 3.36, *p* = .07): all participants were faster to approach rather than avoid alcohol pictures (*t*(59) = 3.09, *p* < .01, *d* = .40) but RTs to approach and avoid control pictures did not differ (*t*(59) = .99, *p* = .33, *d* = .13). At post-test, the picture type × movement × condition interaction was statistically significant (*F*(1,58) = 5.63 *p* = .02). The movement × condition interaction was not significant for control pictures (*F*(1, 58) = .72, *p* = .40), but it was marginally significant for alcohol pictures (*F*(1,58) = 3.92 *p* = .05). Participants in the sham training group were significantly faster to approach rather than avoid alcohol pictures, *t*(29) = −3.43, *p* = .00, *d* = .70. However, this effect was absent in the active training group, as RTs to approach and avoid alcohol pictures were similar, *t*(29) = 1.00, *p* = .32, *d* = .18.

This pattern was confirmed by an analysis of overall ‘approach bias’ scores, which were calculated by computing the speed of avoidance (minus approach) of alcohol pictures and subtracting the speed of avoidance (minus approach) of control pictures, such that positive values indicate a bias for speeded approach of alcohol pictures, and negative values indicate a bias for speeded avoidance of alcohol pictures. The two-way interaction time × condition approached significance (*F*(1, 58) = 3.47, *p* = .07). Groups did not differ on overall bias at pre-test (*t*(58) = .23, *p* = .82, *d* = .06), whereas at post-test, overall alcohol approach bias was smaller in the active training group compared to the sham training group (*t*(58) = 2.37, *p* = .02, *d* = .61). Furthermore, within-group contrasts testing for change over time revealed that, for participants in the active training group, overall bias scores changed from positive at pre-test (M = 23.91 ms) to negative at post-test (M = − 21.57 ms), and this difference approached significance (*t*(29) = 1.97, *p* = .06, *d* = .36). Whereas for participants in the sham training group, overall bias scores were positive at pre-test (M = 18.67 ms), and not significantly different at post-test (M = 31.74 ms) (*t* (29) = .61, *p* = .55, *d* = .11).

### Reaction times before and after inhibitory control training

Go reaction times were analyzed with a mixed design ANOVA, with within-subject factors of time (2: pre-test, post-test) and picture type (2: alcohol, control) and a between-subject factor of condition (2: active training, sham training) (Table 3). There was a statistically significant main effect of picture type (*F*(1,58) = 15.73 *p* < .01) reflecting, on average, slower go RTs on trials with alcohol pictures than neutral pictures. However, the hypothesized time × picture type × condition interaction was not significant (*F*(1,58) = .80 *p =* .37), and there were no other significant main effects or interactions (*Fs* < 2.84, *ps* > .10). Therefore, contrary to hypotheses, ICT that involved pairing alcohol cues with inhibition of responding did not lead to a slowing of go RTs on trials when alcohol cues were presented. We also conducted a supplementary analysis to investigate if RT slowing might be detected by focussing only on responses to alcohol pictures that were used during training, and only on the first few trials of the pre-test and post-test blocks. This analysis did not detect any evidence for RT slowing to alcohol cues after active ICT training. See supplementary materials for details.

**Table 3:** Reaction times (milliseconds) on ‘go’ trials with alcohol and control pictures during the go/no-go (GNG) task

	Active training	Sham control
*Pre-test*		
*Alcohol cues*	*519.68 (54.24)*	*501.64 (52. 49)*
*Control cues*	*518.46 (54.83)*	*491.80 (48.53)*
*Post-test*		
*Alcohol cues*	*521.73 (58.20)*	*506.16 (52.38)*
*Control cues*	*509.85 (53.84)*	*492.45 (45.92)*

Values are shown separately for active training and sham control training groups, and at pre-test (before inhibitory control training) and post-test (after inhibitory control training). Values are mean ± SD

### Automatic evaluations of alcohol pictures

Automatic evaluations of alcohol pictures, assessed with the IAT *d* measure, were analyzed using a 2 × 2 × 2 ANOVA, with a within-subject factor of time (2: pre-test, post-test) and between-subject factors of training type (2: CAT or ICT) and condition (2: active training, sham training) (Table 4). The critical time × training type × condition interaction was not significant (*F*(1116) = 1.78 *p =* .19), and there were no other significant main effects or interactions (*Fs* < .41, *ps* > .52). Therefore, automatic evaluations of alcohol cues did not change from pre-test to post-test after either type of training, contrary to predictions. However, we note that participants held robust associations between alcohol and positive words at both pre-test and post-test, as evidenced by the observation that *d* values were positive and significantly greater than zero (one-sample *t* tests compared to zero; pre-test *t*(119) = 4.41, *p* < .01; post-test *t* (119) = 6.48, *p* < .01).

**Table 4:** Automatic evaluations of alcohol pictures as inferred from participants’ performance on the implicit association task (IAT), at pre-test and post-test

	CAT	Sham CAT	ICT	Sham ICT
*Pre-test*	*.27 (.58)*	*.21 (.73)*	*.21 (.53)*	*.30 (.63)*
*Post-test*	*.20 (.48)*	*.28 (.54)*	*.34 (.44)*	*.30 (.43)*

Positive values indicate stronger associations between alcohol pictures and positively valenced words rather than negatively valenced words. Values are *d measures* (mean ± SD)

## Discussion

The primary finding in the present study was that participants who completed a single session of CAT or ICT consumed less alcohol during a bogus taste test than participants who completed control (‘sham’) versions of these interventions. Most importantly, we observed no significant difference in the magnitude of the effect produced by these two forms of CBM. In addition, and contrary to expectations, we did not observe robust strengthening of alcohol-avoidance or alcohol-inhibition associations after CBM, and neither form of CBM led to devaluation of alcohol-related cues, as inferred from an implicit association task.

Regarding the effects of CBM on alcohol consumption, our findings replicate previous demonstrations of reduced alcohol consumption after a single, brief session of CAT (see Kakoschke et al. 2017a) and ICT (see Allom, Mullan and Hagger 2015; Jones et al. 2016), compared to control CBM. Importantly, the present study is the first head-to-head comparison of these two forms of CBM, and our findings suggest that both are likely to be equally effective for the reduction of alcohol consumption. It is important to note that this was a laboratory investigation of a single session of CBM and we inferred participants’ motivation to drink alcohol based on how much alcohol they consumed during a bogus taste-test (see Jones et al. 2016). The present findings are an important proof of concept, and it is important to investigate their relevance in real-world settings and investigate if multiple sessions of ICT and CAT would prompt comparable reductions in alcohol consumption if delivered to alcohol-dependent patients (see Cristea, Kok and Cuijpers 2016). It would also be of interest to investigate whether a combined intervention would yield larger or more robust effects than either intervention on its own, as suggested by some recent laboratory studies (Kakoschke et al. 2017a, b).

Contrary to hypotheses, we did not observe robust increases in the strength of alcohol-avoidance associations in participants who completed a single session of CAT. However, we did observe a notable trend in that all participants were faster to approach alcohol rather than avoid alcohol pictures before CBM; after CAT, this ‘approach bias’ was maintained in the sham training (control) group, but it was eliminated in the group that received active CAT. Closer inspection of the previous literature demonstrates that changes in alcohol-avoidance associations after CAT are often observed (Wiers et al. 2010, 2011; Eberl et al. 2013, 2014; Sharbanee et al. 2014; Gladwin et al. 2015), but there are notable exceptions, even in studies in which CAT led to changes in brain activation during exposure to alcohol cues (Wiers et al. 2015) or improved abstinence rates after treatment (Manning et al. 2016). One interpretation for these findings is that there are methodological limitations to tasks that are used to measure alcohol-avoidance associations, such as the approach-avoidance IAT (used in some of the above studies) and slowing of reaction times during the irrelevant-feature AAT (used in the present study). For example, the irrelevant-feature AAT has poor internal reliability and predictive validity (in comparison to alternative tasks such as the relevant-feature stimulus-response compatibility task; see Kersbergen et al. 2015), which may render it relatively insensitive for the purposes of assessing changes in alcohol-avoidance associations that are expected to arise after CAT.

Similarly, and again contrary to hypotheses, we did not observe any slowing of reaction time to alcohol cues, which would indicate the formation of alcohol-inhibition (or ‘stopping’) associations, after ICT. Numerous laboratory studies that used arbitrary stimuli (Verbruggen and Logan 2008b, 2009; Lenartowicz et al. 2011; Verbruggen et al. 2014), and indeed some studies that used alcohol-related stimuli (Jones and Field 2013; Noël et al. 2016) have demonstrated the robustness of these stop-learning effects, so in a sense, our findings are surprising. However, other studies, particularly those that investigated ICT in applied domains, did not demonstrate the predicted formation of cue-stopping associations, in some cases even after multiple sessions of ICT (Houben et al. 2012; Lawrence et al. 2015). The reasons for these discrepant findings are unclear; however, recent laboratory studies suggest that stop-learning effects may be sensitive to a number of factors including task instructions (Best et al. 2015), the presence of an executive setting (i.e., a setting in which participants might be required to inhibit; Chiu and Aron 2014) or individual differences in the motivational response to the stimuli used (Stice et al. 2016). Alternatively, and in common with our discussion of the internal reliability of reaction time measures obtained from the irrelevant AAT (above), it is possible that reaction times on ‘go’ trials are not sufficiently reliable or sensitive to detect changes that arise as a result of a brief session of ICT. Further work is required to identify a reliable measure of cue-stopping associations that is sensitive to the effects of ICT.

Furthermore, we observed no effect of either form of CBM on devaluation of alcohol cues, as inferred from participants’ performance on a bipolar implicit association test (IAT). This suggests that the reduction in alcohol consumption after both CAT and ICT cannot be attributed to changes in automatic evaluations of alcohol pictures. We opted to use the IAT to measure devaluation on the basis of two previous studies which used the same measure to demonstrate that a single session of ICT led to robust changes in automatic evaluations of alcohol pictures (Houben et al. 2011, 2012). Therefore, we failed to replicate these earlier findings, as did another recent study which also investigated the effects of a single session of ICT on the same task (Bowley et al. 2013). There are a number of possible explanations for why the effects of CBM on stimulus devaluation as inferred from IAT performance do not appear to be robust across studies. First, as a reaction time measure, it may be subject to similar confounds that complicate interpretation of changes in the speed of avoidance or slowed responding to target stimuli after CAT and ICT, respectively, as discussed above. Second, the IAT may not be sufficiently sensitive to detect changes in automatic stimulus evaluations after a brief session of CBM (Woud et al. 2013a, b; Becker et al. 2015). Third, devaluation effects may be more robust when different measures of stimulus valuation such as subjective ratings or auction tasks are used instead of the IAT (Veling et al. 2008; Ferrey et al. 2012; Wessel et al. 2014; Lawrence et al. 2015 Veling et al. 2017; although see Wiers et al. 2015).

Our study has additional limitations, in addition to some notable strengths. In common with most other laboratory CBM studies, group allocation was single rather than double blinded: the experimenter was aware of group allocations, but participants were not. This increases the risk of bias in such studies (see Cristea et al. 2016). However, participants were led to believe that there was no experimental manipulation in the study, and indeed, their responses during formal debriefing indicated that the vast majority of participants (across all groups) believed this cover story, and only a tiny minority (6 out of 120 participants; 5%) developed awareness of the intended purpose of CBM (see supplementary file). Therefore, it seems unlikely that demand characteristics could account for the effects of CAT and ICT on alcohol consumption. Additionally, our sample was predominantly female and we did not record participants’ ethnicity; however, supplementary analyses confirmed that participant sex did not moderate any of the effects. Our study also has strengths, including the large sample size and the use of a 50:50 contingency between alcohol pictures and avoidance (or inhibition) in the sham (control) conditions. This type of control manipulation helps to resolve ambiguity regarding interpretation of findings from previous studies that compared CBM with control conditions that attempted to increase (rather than extinguish or reverse) cognitive biases (Wiers et al. 2010b; Houben et al. 2012; Kakoschke et al. 2017a), which could have inflated the apparent effect size of CBM by increasing value of appetitive stimuli in these ‘control’ conditions (Schonberg et al. 2014).

To conclude, we demonstrated that a single, brief session of CAT or ICT yielded equivalent reductions in alcohol consumption in the laboratory. However, neither form of CBM resulted in robust strengthening of alcohol-avoidance or alcohol-inhibition associations, and neither led to devaluation of alcohol-related cues. Further research is required to identify the psychological mechanisms that underlie the effects of these forms of CBM on alcohol consumption.

## Electronic supplementary material

ESM 1 (DOC 125 kb)
