# Supplementary material for: Cue avoidance training and inhibitory control training for the reduction of alcohol consumption: a comparison of effectiveness and investigation of their mechanisms of action
Source: Psychopharmacology (Berl). 2017 May 27;234(16):2489–98. doi: 10.1007/s00213-017-4639-0 (PMC5537323; doi:10.1007/s00213-017-4639-0)
Supplement: Supplementary file 1 — (DOC 125 kb) [file 213_2017_4639_MOESM1_ESM.doc]

**Supplementary materials**

**Introduction**

We report a detailed description of the implicit association test (IAT). We also report findings related to a number of secondary hypotheses that were not covered in the primary manuscript. In particular, we investigated (a) whether effects of CAT and ICT on alcohol-approach and alcohol-inhibition associations would generalize to novel stimuli that were not used during training blocks (see Wiers et al., 2010); (b) if participants’ awareness of the purpose of CAT or ICT, or their awareness of the experimental hypotheses, would moderate the effects of CAT or ICT on alcohol consumption during the taste test (see Field et al., 2007); (c) if post-training performance on any of the cognitive tasks was associated with individual differences in beer or soda consumption during the taste test; and (d) if any of the analyses reported in the main manuscript were moderated by participant sex. We also report participants’ accuracy on the tasks during training and test blocks.

**Methods**

*Description of the Bipolar Alcohol Valence IAT.*

Participants were instructed to classify stimuli into two target categories (alcohol or stationery pictures, 10 pictures each) and two attribute categories (positive or negative words, 6 each), by responding on one of two different response keys (left and right) as quickly as possible. The modified version used in the present study was adapted from Houben et al. (2012), the only differences regarded the use of different neutral stimuli (instead of empty glasses we adopted photographs of stationery items), and we included 10 (rather than 6) alcohol and stationery pictures in order to match the number of pictures used in the assessment blocks of the two CBM interventions. The attribute words were the same as those used in the earlier study.

The underlying idea is that the simultaneous classification of targets and attributes is easier and faster when the target and the attribute are strongly associated. If a participant is faster to respond when alcohol pictures and positive words share a response key compared to when alcohol pictures and negative words share a response key, this indicates that alcohol-positive associations are stronger than alcohol-negative associations for that participant.

The IAT comprised seven blocks. In the first two blocks (practice blocks, 24 trials each) participants were asked to practice the target (alcohol vs. stationery) categorization and then the attribute (pleasant vs. unpleasant words) categorization using two response keys (left and right). The third block (also 24 trials) was a practice combination block in which participants pressed one key for one target category *or* one attribute category (e.g. alcohol pictures *or* positive words), and a different key for the other target or attribute category (e.g. stationery pictures *or* negative words). The fourth block (48 trials) was the test combination block in which participants continued to categorize the pictures and words using the same keys as in block 3. Block 5 (48 trials) was another practice categorisation block, with the key difference that the mapping of response keys to alcohol and neutral stimuli was reversed from that applied during block 1. Block 6 (24 trials) was a reversed practice combination block in which participants practiced the opposite combination of target and attribute categories present in block four (e.g. responding on one key for alcohol or unpleasant words and a different key for neutral or pleasant words). Finally, block 7 tested the combination they just practiced (reversed test block, 48 trials). Response key assignment and the order of the combined sorting conditions (in blocks 3, 4, 6 and 7) were counterbalanced across participants.

IAT effects were calculated with the *d measure* (Greenwald et al., 2003). Response latencies less than 300ms or more than 10000ms were discarded. Error latencies were replaced by the block mean + 2 standard deviations. Mean RTs were calculated separately for both sub-blocks of the combination task (block 3 and 6 and block 4 and 7). The *d* measure was then calculated as the standardized difference between these two RTs divided by the standard deviation of RTs in both blocks. A stronger positive *d* score indicates stronger associations between alcohol cues and positive valence.

*CAT and ICT generalization effects: Trained versus novel picture sets.*

In order to test if effects of CAT and ICT would generalize from the specific stimuli used during training to novel alcohol-related stimuli (that were not used during training), the pre-test and post-test assessment blocks contained 10 additional pairs of alcohol-related and control stimuli that were not used during the training block. To investigate generalization effects, we repeated the analysis of reaction time data (as reported in the manuscript), but with an additional within-subjects factor of picture set (trained vs. novel).

*Participants’ awareness of the study aims and hypotheses*

We probed participants’ awareness of the intended purpose of CAT and ICT and of the taste test, in addition to their awareness of the overall aims of the study. To achieve this we used a combination of open-ended and multiple choice questions based on those used in previous research (Field et al., 2007; Jones and Field, 2013). First, participants provided a written response to an open-ended question ‘*What was the general purpose of the experiment*’? The second question was a multiple choice question which assessed participants’ awareness of the intended purpose of the CAT or ICT training. The question was phrased as ‘*The computer task where you had to respond by… moving the joystick (CAT groups only)… (or) pressing the space bar to letters p or f (ICT groups only )…, was designed to*…..’? There were five response options: a. *Train me to think more quickly*; b. *Measure how quickly I can categorise things*; c. *Measure my ability to control myself when I think of alcohol;* d. *Teach me to control myself when I think of alcohol*; e. *I do not know what this task was measuring*. The final question assessed participants’ awareness of the purpose of the bogus taste test, and was worded: ‘*The purpose of the Taste-Test was to….’:* There were five response options: *a. Measure my liking for each drink*; b. *Measure how much I wanted to drink alcohol* (participants who selected this option were classed as aware of the purpose of the taste test); c. *Measure my thirst*; d. *Find out which drink I preferred*; e. *I do not know the purpose of this task*).

**Results**

*Do effects of CBM generalize to pictures that were not used during the training block?*

*Effects of CAT on trained vs. novel pictures (Table S1a).*

Reaction times were subjected to a 2 X 2 X 2 X 2 X 2 mixed design ANOVA, with within-subject factors of Time (2: pre-test, post-test), Picture type (2: alcohol, control), Movement (2: approach, avoidance), Picture Set (2: trained pictures, novel pictures) and a between-subject factor of Condition (2: active training, sham training). To avoid duplication with the primary results section, only significant main effects or interactions that involve Picture Set are reported here.

There was a significant main effect of Picture Set (F (1, 58) = 23.20 *p* < .01), a significant Time X Picture Set interaction (F (1, 58) = 4. 35 *p* = .04), and trends for a Movement X Picture Set X Condition interaction (F (1, 58) = 3.04 *p* =.09) and a Time X Movement X Picture Set X Condition (F (1, 58) = 3.83 *p* =.06) interaction. These main effects and interactions reflect the observation that participants were generally faster to respond to trained pictures rather than novel pictures, and this difference was particularly noticeable (1) at post-test, compared to pre-test, (2) for approach movements rather than avoidance movements, and (3) both of these differences were slightly more pronounced in the active training group compared to the sham training group. Details of these post-hoc tests are available on request. Importantly, the five-way interaction Time x Movement x Picture Set x Picture Type x Condition interaction was not statistically significant (F (1, 58 = .63, *p* = .43). This demonstrates that, although there were noticeable differences between reaction times to trained and novel pictures, this pattern did not differ for alcohol and control pictures by experimental group and therefore the effects of CAT on reaction times to alcohol and control pictures (as reported in the main manuscript) were not different for stimuli that were used during training, or novel stimuli.

*Effects of ICT on trained vs. novel pictures (Table S1b).*

Reaction times on Go trials were analyzed with a 2 X 2 X 2 X 2 mixed design ANOVA, with within-subject factors of Time (2: pre-test, post-test), Picture type (2: alcohol, control), Picture set (2: trained pictures, novel pictures) and a between-subjects factor of Condition (2: active training, sham training). To avoid duplication with the primary results section, only main effects or interactions that involve Picture Set are reported here.

The main effect of Picture Set was statistically significant, (F (1, 58) = 4.02 *p* = .05) and it was subsumed under interactions between Time X Picture set (F (1, 58) = 5.57 *p* = .02) and Time X Picture type X Picture Set (F (1, 58) = 4.66 *p* = .04) were significant. These main effects and interactions arose because participants were generally faster to respond to trained pictures rather than novel pictures, a difference that was particularly evident (1) at post-test compared to pre-test; (2) for alcohol pictures compared to control pictures, and (3) for participants in the active training group compared to participants in the sham training group. Details of these post-hoc tests are available on request. Importantly, there were no other significant main effects or interactions involving Picture Set (Picture Type x Picture Set x Condition, F(1, 58) = .18, p = .68; Picture Type x Picture Set x Condition x Time, F(1, 58) = .37, p = .54).

In response to a helpful suggestion from an anonymous reviewer that participants may rapidly habituate to No-Go paired stimuli during test blocks, and this effect may have been obscured by the incorporation of novel stimuli, we performed an additional analysis to investigate reaction times on Go trials, but we limited this analysis to trained stimuli during the first half of trials in the pre- and post-test blocks. These reaction times were analysed with a 2 X 2 X 2 ANOVA, with within-subject factors of Time (2: pre-test, post-test), Picture type (2: alcohol, control) and a between-subjects factor of Condition (2: active training, sham training). The three way interaction was not statistically significant (*F*(1,58) = 1.31 *p* = .26) and there were no other significant main effects or interactions (*F* < 1.86, *p* > .18). These analyses demonstrate that, although there were noticeable differences between reaction times to trained and novel pictures, this pattern did not differ for alcohol and control pictures by experimental group, and this was also the case when analysis was restricted to the first half of trials in each test block.

*Participants’ awareness of the study aims and hypotheses (Table S2)*

The first question in the funnelled debriefing required participants to identify what they thought was the main aim of the study. Their responses revealed that the vast majority of participants (116; 97 %) were unaware of the aims and hypotheses. Answers to this open-ended question were varied, but recurring themes were advertising, individual differences in liking of tastes of different drinks, and how individual differences in alcohol consumption may influence cognitive performance.

Participants’ responses to the next (multiple choice) question are shown in Table S2. It is evident that the majority of participants believed the cover story that the study was an investigation of the relationship between cognitive performance and individual differences in drinking habits, because the majority thought that the purpose of the training task was to ‘measure my ability to control myself when I think of alcohol’ (46%) or ‘measure how quickly I can categorise things’ (36%). Only six participants (5%; 5 in active training groups, 1 in sham training group) thought that the purpose of the training task was to ‘teach me to control myself when I think of alcohol’. Although it appears that participants in the active training groups were more likely to select this option than participants in the sham training groups, a Chi Square test confirmed that there was no significant relationship between group allocation and the response option selected *χ^2^*(12) = 14.49, *p* = .27.

Participants’ responses to the final question revealed that the majority were aware of the real purpose of the Taste-Test, with 63 participants (53 % of the sample) correctly identifying that this task was a measure of their motivation to drink alcohol. To explore the influence of this factor, we repeated the analysis of taste test data (see Figure 1 in the main manuscript) with the addition of awareness (2: aware, unaware) as an additional between-subjects factor. This analysis revealed that the main effect of drink type (F (1,112) = 11.91 *p* < .01) and the 2-way interaction drink type X condition (F (1,112) = 20.45 *p* < .01) that were reported in the main manuscript, remained statistically significant. Importantly, the 3-way interaction drink type x condition x awareness was not significant (F (1, 112) = .26 *p* = .61) and there were no other significant interactions or main effects (Fs < 2.56, *p*s > .11). Therefore, participants’ awareness of the purpose of the taste test did not influence the primary findings.

*Response errors (Table S3)*

Participants made very few errors on the Go/No-Go and Approach Avoidance tasks during pre-test, training and post-test blocks. Given the skewed distribution of error data, these were not formally analysed.

*Correlations between task performance and consumption during the taste test (Table S4).*

To investigate if individual differences in performance on the cognitive tasks at post-test were associated with individual differences in beer or soda consumption, we correlated drink consumption (as a percentage of fluid available) with the IAT *d* measure, and with alcohol approach bias (CAT groups only) and alcohol inhibition bias (ICT groups only). The latter bias scores were computed on the basis of both reaction time and error data. These correlations are reported in Table S4, initially for the sample as a whole and then separately for each of the four experimental groups. After correction for multiple comparisons, none of these correlations were statistically significant.

*Sex differences*

In order to investigate if participant sex moderated any of the primary findings reported in the main manuscript, we repeated all primary analyses after adding sex as an additional between-subjects factor. These analyses confirmed that sex did not moderate the effects reported here: there were no interactions involving sex and either condition or training type, and the findings reported in the manuscript were unaffected.

**Supplementary discussion**

In line with previous CBM work, we demonstrated that effects of CBM on reaction times to alcohol cues were not noticeably different for stimuli that were used during training compared to novel alcohol stimuli (Wiers et al., 2010). In general, reaction times for pictures that had been used during CBM were faster than reaction times to novel pictures, however the absence of interactions with experimental condition, picture type and time suggests that this did not affect generalization of effects of CBM from trained to novel stimuli. Furthermore, in line with findings from previous studies (Wiers et al., 2010; Houben and Jansen, 2011) participants were not aware of the overall aim of the study. In general, it seemed that most participants believed the cover story that they were provided with. Additionally, we observed no significant correlations between individual differences on the cognitive tasks at post-test, and beer or soda consumption.

A limitation is that we can only indirectly infer that individuals in the active training groups were not aware of the contingencies that were applied during CBM, because our awareness questions assessed their awareness of the purpose of CBM rather than the contingencies that were applied during training. Some previous ICT studies have demonstrated that most participants become aware of the contingency between appetitive cues and the requirement to inhibit (Lawrence et al., 2015a; Lawrence et al., 2015b), as inferred from expectations of having to stop when those cues are encountered (Best et al., 2015). In the light of some recent findings, future studies could investigate the effects of providing participants with explicit information about training contingencies before they receive CBM (Van Dessel et al., 2015, 2016; Van Dessel, De Houwer and Gast, 2016).

Finally, approximately half of our participants were aware of the real purpose of the taste test. However, participant awareness did not affect our primary findings, because participants who received active CBM drank less alcohol than participants who received sham (control) CBM, regardless of their awareness of the purpose of the taste test (see Jones, Button, et al., 2016).

**References**

Field M, Duka T, Eastwood B, Child R, Santarcangelo M, Gayton M (2007) Experimental manipulation of attentional biases in heavy drinkers: Do the effects generalise? Psychopharmacology, 192: 593–608.

Jones A, Button E, Rose A K, Robinson E, Christiansen P, Di Lemma L C D, Field M (2016) The ad-libitum alcohol “taste test”: Secondary analyses of potential confounds and construct validity. Psychopharmacology, 233: 917-924.

Lawrence N S, Verbruggen F, Morrison S, Adams R C, Chambers C D (2015b) Stopping to food can reduce intake. Effects of stimulus-specificity and individual differences in dietary restraint. Appetite, 85: 91-103.

Van Dessel P, De Houwer J, Gast A (2016) Approach–avoidance training effects are moderated by awareness of stimulus–action contingencies. Personality and Social Psychology Bulletin, 42: 81–93.

Van Dessel P, De Houwer J, Gast A, Smith C T (2015) Instruction-based approach-avoidance effects: Changing stimulus evaluation via the mere instruction to approach or avoid stimuli. Experimental Psychology, 62: 161–169.

Van Dessel P, De Houwer J, Gast A, Smith C T, De Schryver M (2016) Instructing implicit processes: When instructions to approach or avoid influence implicit but not explicit evaluation. Journal of Experimental Social Psychology, 63: 1–9.

Table S1a. Reaction times (milliseconds) to approach and avoid alcohol and control pictures during the approach-avoidance task (AAT). Values are shown separately for active training and sham training groups, and at pre-test (before cue avoidance training) and post-test (after cue avoidance training), respectively for trained and untrained picture sets. Values are mean ± SD.

Active Training Sham Control

*Pre-test*

*Approach Alcohol Trained 739.49 (126.64) 745.26 (137.59)*

*Avoid Alcohol Trained 803.62 (155.96) 754.27 (118.53)*

*Approach Alcohol Novel 775.32 (171.80) 741.54 (128.32)*

*Avoid Alcohol Novel 793.58 (173.44) 773.88 (125.15)*

*Approach Control Trained 758.60 (161.68) 768.29 (143. 59)*

*Avoid Control Trained 794.49 (177.56) 768.70 (134.28)*

*Approach Control Novel 784.86 (190.52) 770.63 (153.19)*

*Avoid Control Novel 784.20 (163.01) 774.17 (142.46)*

*Post-test*

*Approach Alcohol Trained 743.46 (145.59) 733.36 (165. 36)*

*Avoid Alcohol Trained 752.12 (142.25) 786.50 (180.77)*

*Approach Alcohol Novel 773.81 (160.94) 762.87 (138.61)*

*Avoid Alcohol Novel 783.53 (147.25) 808.67 (196.06)*

*Approach Control Trained 728.10 (129.10) 748.86 (174.65)*

*Avoid Control Trained 772.37 (166.16) 772.35 (176.58)*

*Approach Control Novel 770.07 (149.43) 780.76 (188.98)*

*Avoid Control Novel 795.29 (163.78) 792.35 (190.40)*

Table S1b. Reaction times (milliseconds) on ‘Go’ trials with alcohol and control pictures during the Go / No-Go (GNG) task. Values are shown separately for active training and sham training groups, and at pre-test (before inhibitory control training) and post-test (after inhibitory control training), respectively for trained and untrained picture sets. Values are mean ± SD.

Active Training Sham Control

*Pre-test*

*Alcohol Trained 515.99 (55.27) 497.95 (50.91)*

*Control Trained 520.43 (53.70) 498.98 (50.16)*

*Alcohol Novel 525.15 (61. 95) 502.16 (52.81)*

*Control Novel 514.71 (55.40) 490.35 (53.78)*

*Post-test*

*Alcohol Trained 521.24 (62.17) 469.35 (50.66)*

*Control Trained 505.29 (52.59) 488.89 (56.39)*

*Alcohol Novel 522.25 (68.48) 509.17 (53.97)*

*Control Novel 515.33 (61.26) 502.75 (55.06)*

Table S2. Frequencies of participants’ responses to the question that probes their awareness of the purpose of CBM.

CAT Sham CAT ICT Sham ICT

**a.** *Train me to think more quickly* 1 1 1 2

**b.** *Measure how quickly I can categorise things* 10 13 10 10

**c.** *Measure my ability to control myself when I think of alcohol* 14 14 16 11

**d.** *Teach me to control myself when I think of alcohol* 3 1 2 0

**e.** *I do not know what this task was measuring* 2 1 1 7

T

Table S3. Response errors. Values are shown separately for active training and sham training groups, and at pre-test (before training), during training and post-test (after training), respectively. Values are means (SD in brackets)

                Active Training      Sham Control

*Pre-test (80 trials)*

*AAT errors*  *4.53 (8.08) 2.83 (2.48)*

*No-Go errors (from 40 No-Go trials) .40 (.89)                    .77 (.94)*

*Training (480 trials)*

*AAT errors 19.73 (28.76)      13.03 (12.34)*

No-Go errors (from 240 No-Go trials) *2.77 (2.30)     4.10 (4.41)*

*Post-test (80 trials)*

*AAT errors 4.37 (6.85)       2.53 (2.64)*

*No-Go errors (from 40 No-Go trials)  .90 (1.09)     .77 (1.19)*

Supplementary Table S4. Correlation matrix between alcohol (1.) and soda (2.) consumption during the taste test and post-training bias scores for the sample as a whole and stratified by each experimental group. Values are….

Whole sample CAT Sham CAT ICT Sham ICT

Variables **1. 2. 1. 2. 1. 2. 1. 2. 1. 2.**

*IAT D-measure       .01 .10          - .05  -.01 .03 .05 .02 .29 .02 .15*

*AAT bias (RTs)    .14  .08 .16 .06 -.04 .18 / / / /*

*AAT bias (Errors)   .20   -.03 .07 -.06 .26 .10 / / / /*

*Go / No-Go Go RT bias .07     .08      /  / / / .14 .11 .00 .07*

*Go/ No-Go No-Go error bias .16 .22          /  /  / / .26 -.07 -.09 -.36*
